# Stem Cell-Based Therapies for Parkinson Disease

**DOI:** 10.3390/ijms21218060

**Published:** 2020-10-29

**Authors:** Zhaohui Liu, Hoi-Hung Cheung

**Affiliations:** 1 Faculty of Medicine, School of Biomedical Sciences, The Chinese University of Hong Kong, Hong Kong, China; Kiyaliu@cuhk.edu.hk; 2 Key Laboratory for Regenerative Medicine, Ministry of Education (Shenzhen Base), Shenzhen Research Institute, The Chinese University of Hong Kong, Shenzhen 518057, China

**Keywords:** Parkinson disease, stem cell, cell-based therapy, iPSC, dopamine, cell transplantation

## Abstract

Parkinson disease (PD) is a neurological movement disorder resulting primarily from damage to and degeneration of the nigrostriatal dopaminergic pathway. The pathway consists of neural populations in the substantia nigra that project to the striatum of the brain where they release dopamine. Diagnosis of PD is based on the presence of impaired motor features such as asymmetric or unilateral resting tremor, bradykinesia, and rigidity. Nonmotor features including cognitive impairment, sleep disorders, and autonomic dysfunction are also present. No cure for PD has been discovered, and treatment strategies focus on symptomatic management through restoration of dopaminergic activity. However, proposed cell replacement therapies are promising because midbrain dopaminergic neurons have been shown to restore dopaminergic neurotransmission and functionally rescue the dopamine-depleted striatum. In this review, we summarize our current understanding of the molecular pathogenesis of neurodegeneration in PD and discuss the development of new therapeutic strategies that have led to the initiation of exploratory clinical trials. We focus on the applications of stem cells for the treatment of PD and discuss how stem cell research has contributed to an understanding of PD, predicted the efficacy of novel neuroprotective therapeutics, and highlighted what we believe to be the critical areas for future research.

## 1. Introduction

Parkinson disease (PD) is the second most common neurodegenerative disorder after Alzheimer disease. PD affects more than six million people globally, predominantly those over the age of 65 years. The mean age of PD onset is 55 years, and the major risk factor for the development of PD is aging [1]. Approximately 10% of patients with PD have young-onset PD, defined as a diagnosis between 21 and 50 years of age, [2], more likely to be familial or genetic [3]. For later-onset PD, patients are usually diagnosed over 70 years of age [4].

The clinical presentation of PD is motor dysfunction, which is characterized by bradykinesia, muscle rigidity, resting tremor, and postural instability. Most neurodegenerative processes related to PD are driven by the accumulation of pathological α-synuclein (α-syn) [5], which is a presynaptic neuronal protein that aggregates to form Lewy bodies (LBs) and Lewy neurites (LNs) in the nervous system [6,7].

Motor impairment in patients with PD is common and increases markedly with age. The most common symptom of PD is tremor, which usually occurs at rest but decreases with voluntary movement [8]. Additional nonmotor symptoms include hyposmia, constipation, anxiety, depression, orthostatic hypotension, urinary dysfunction, rapid eye movement sleep behavior disorder, and cognitive dysfunction. A proportion of PD patients also develop mental health problems, such as depression and dementia. PD with dementia often develops years after the onset of motor dysfunction and is estimated in 30–80% of affected individuals [9,10]. 

The exact cause of PD is still unknown. Advanced age is known as the greatest risk factor for PD. Aging can cause a cascade of stressors within the substantia nigra (SN), thus weakening neurons and their ability to respond to further insults [11,12]. Both environmental and genetic factors are recognized to affect disease risk and progression. Only approximately 10% of PD cases can be directly attributed to genetic factors [13]. Regarding environmental factors, vascular insults to the brain, repeated head trauma, neuroleptic drugs, exposure to pesticides, smoking, caffeine intake, and manganese toxicity are all reported to increase the risk of developing parkinsonism [14].

In this paper, we briefly review our current understanding of the pathogenesis of PD and the application of different animal models for studying the disease, and we discuss current treatments. We emphasize the latest advancements in applying stem cell therapy for treating PD.

## 2. Pathophysiology

**Figure 1:**
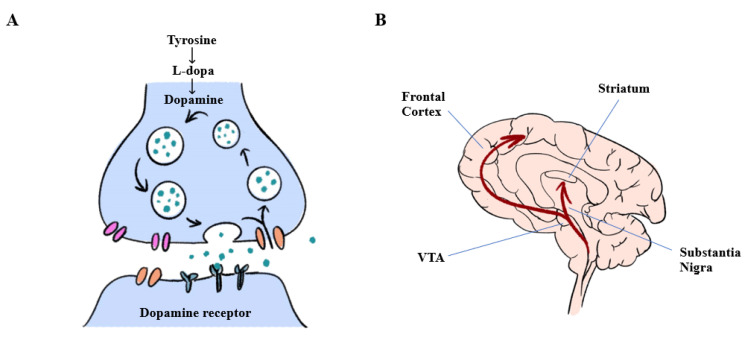
Pathway of dopamine biosynthesis in dopaminergic neurons and dopamine release in the brain. (**A**) Enzymes for the biosynthesis of dopamine are specifically expressed in midbrain dopaminergic neurons. Tyrosine hydroxylase (TH) converts tyrosine to L-dopa, which is further converted to dopamine by aromatic L-amino acid decarboxylase. (**B**) Dopamine pathways in the central nervous system. VTA: ventral tegmental area.

With regard to pathology, loss of dopaminergic (DA) neurons in the SN pars compacta (SNpc) in the midbrain and the existence of cytoplasmic protein aggregates known as LBs in the remaining DA cells are the hallmarks of PD (Figure 1). SNpc neurons form the nigrostriatal DA pathway; thus, loss of SNpc neurons leads to the striatal dopamine deficiency responsible for the major symptoms of PD. Hence, replenishment of striatal dopamine through the oral administration of the dopamine precursor levodopa (L-DOPA) alleviates most of these symptoms [6].

Degeneration of DA neurons results in a threshold decrease of approximately 80% dopamine in the striatum, leading to the emergence of motor symptoms [15]. Mechanically, the death of DA neurons has been linked to mitochondrial dysfunction, oxidative stress, neuroinflammation, and insufficient autophagic or proteasomal protein degradation. With the loss of nigrostriatal DA structures and functioning, PD also affects many other areas of the central nervous system [16].

α-syn is a presynaptic nerve terminal protein of 140 amino acids that constitutes a major component of LBs and LNs in sporadic PD [17]. Native α-syn appears unfolded and represents approximately 1% of cytosolic protein in the brain. Results from α-syn knockout mice suggest a role for α-syn as a presynaptic activity-dependent negative regulator of dopamine release [18]. Knockin and overexpression of α-syn mutants seem to be especially toxic to DA neurons [19]. These outcomes support the conjecture that α-syn plays an important role in the nigrostriatal dopamine pathway; rodent-based models using overexpressing mutated α-syn are required to further investigate the pathogenesis of PD. In sporadic PD, neuropathological assessment of the distribution of misfolded α-syn indicates that it is a global nervous system disorder, with DA neurons becoming affected in the mid-stage of the disease course [20]. Overexpression of α-syn has been shown to reduce DA release in transgenic, pharmacological, and toxin-based models [21].

## 3. Animal Models of PD

### 3.1. Neurotoxin-Induced PD Models

In many animal studies, PD can be partially mimicked in experimental animals by exposing them to chemicals used as pesticides, insecticides, and herbicides [22]. Such chemicals are believed to be a direct cause of parkinsonism in humans. Neurotoxin-induced PD models have been well developed for rodents and nonhuman primates. Common toxin-induced animal models include stereotaxic injections of 6-hydroxydopamine (6-OHDA), 1-methyl-4-phenyl-1,2,5,6-tetrahydropyridine (MPTP), and α-syn preformed fibrils (PFFs) [23,24]. 

6-OHDA is a synthetic neurotoxic compound used by researchers to selectively destroy DA and noradrenergic neurons in the brain. As an analog of dopamine, 6-OHDA can be taken up into DA neurons by dopamine transporters (DATs). The metabolism of 6-OHDA generates harmful free radicals that cause oxidative stress and mitochondrial dysfunction, leading to neuronal cell death, neuroinflammation, and neurodegeneration. Since 6-OHDA cannot cross the blood–brain barrier, systemic administration fails to induce parkinsonism [25]. 6-OHDA must be injected into the SN, medial forebrain bundle (MFB), or striatum directly [26]. The pattern of DA loss in animals bearing a full lesion (>90%) is found in both 6-OHDA-induced mice and rats.

MPTP was once an industrial chemical and its misuse in humans results in severe motor dysfunction that closely resembles an advanced stage of PD [27]. When injected into animals, MPTP is taken up by astrocytes and metabolized to 1-methyl-4-phenylpyridinium (MPP+), a neurotoxin that impairs electron transport in mitochondria, resulting in the accumulation of ionizing free radicals. Because DAT has a high affinity to MPP+, MPTP can selectively kill DA neurons, giving rise to nigrostriatal pathway deficits. MPTP causes a greater loss of DA neurons in the SN than in the ventral tegmental area or retrorubral field [28]. However, continuous low-dose administration of MPTP mimics more progressive behavioral changes and inclusion formation characteristics of PD [29]. These models can provide gross specimens to mimic DA neuron loss in the SN and damaged dopamine expression [30]. However, they have been criticized because the rate of neurodegeneration is far greater than that in patients with PD. Moreover, neither the 6-OHDA nor the MPTP model produces LB-like inclusions in the nigrostriatal pathway.

PFF derived from the aggregation of recombinant α-syn monomers can initiate the formation of α-syn pathogenic inclusions in nontransgenic or wild-type neurons from mice as well as the propagation of pathological α-syn from one neuron to another [31,32]. In both mice and rat PFF models, introduction of α-syn inclusions in the striatum or MFB caused neuronal dysfunction, mitochondrial damage, and eventually retrograde degeneration of nigrostriatal DA neurons [33]. The most appropriate use of animal models in preclinical research is to assess whether a putative therapy reduces or clears α-syn inclusions in neurons [24].

### 3.2. Transgenic PD Models

Increasing evidence suggests that genetics plays a major role in the etiology of PD, as some genes are linked to the development of rare forms of PD [34]. Both rare and common genetic variants contribute to disease risk, onset, and progression. Mutations in more than 20 genes have been associated with the disease. Monogenic mutations in autosomal dominant genes (e.g., *SNCA*, *LRRK2*, *POLG*) and recessive genes (e.g., *FBXO7*, *PINK1*, *PRKN*, *PARK7*) have been identified [35]. An in-depth discussion of the genetic architecture of PD can be found in another recent review [36]. Many transgenic animal models of PD are generated based on the identified monogenic PD genes. Most genetic models have only been effective at reproducing some hallmarks of PD [37].

Mutations in the *SNCA* gene (encoding α-syn) cause autosomal dominant PD with characteristic LB pathology. LB-associated neural degeneration typically occurs in the absence of any measurable loss of DA neurons. α-syn transgenic animals exhibited robust non-DA deficits including anxiety, gastrointestinal dysfunction, and non–DA-related motoric dysfunction [38]. 

Overexpression of autosomal dominant mutant genes such as *SNCA* (A53T, A30P α-syn) and *LRRK2* (leucine rich repeat kinase 2) (G2019S, R1441C) is induced to replicate familiar PD [39], whereas knockout, knockin, or knockdown of autosomal recessive genes such as *Parkin*, *DJ-1*, and *PINK1* is also performed in rodents to replicate other familiar PD. Genetic mutations in other pathways such as glucocerebrosidase (*GBA*) (E326K and N370S), which is involved in the lysosomal and trafficking pathways, are also known to cause PD [40]. Among these genetic risk factors, the gene *LRRK2* plays a prominent role because the *LRRK2* locus harbors one of the most common polymorphisms associated with PD [41].

A missense mutation of human *SNCA* was the first such mutation to be linked to late-onset familial parkinsonism [42]. The development of nonmotor features correlates with the *SNCA* gene copy number as well as gene and protein expression [43]. Loss-of-function mutations in *PINK1* and *PRKN* are the commonly known causes of autosomal recessive and early onset PD [44,45], suggesting a defect in the mitochondrion and mitophagy functions. A major genetic risk factor for PD is the mutation in the *GBA* gene that encodes glucocerebrosidase, an enzyme linked to Gaucher disease [40]. *GBA* mutation is associated with a typical phenotype of PD termed dementia with LB. Additionally, a pathogenic link exists between GBA and α-syn [46]. The enzymatic activity of glucocerebrosidase is critical to the prevention of α-syn accumulation in patients with prodromal PD [38]. A disease-causing mutation in *LRRK2*, which plays a major causal role in the inheritance of PD, leads to enhanced activity of this kinase and neurodegeneration [47].

## 4. Treatments

No cure for PD has been discovered. Pharmacotherapy for alleviating motor symptoms mostly aims at restoring striatal dopamine tone using dopamine agonists, monoamine oxidase (MAO)-B inhibitors, and L-DOPA plus carbidopa. Dopamine is synthesized by the conversion of L-tyrosine to L-DOPA by tyrosine hydroxylase (TH) and the subsequent decarboxylation of L-DOPA by dopa decarboxylase [48]. DA is metabolized by intraneuronal MAO-A and by glial MAO-A and MAO-B [49]. Dopamine replacement therapy requires the use of L-DOPA because dopamine cannot cross the blood–brain barrier. Once L-DOPA has reached the brain, it is converted to dopamine by the terminals of the surviving nigrostriatal neurons and probably also by the microglia and serotonergic neurons [50]. 

Medication helps to manage problems associated with motor functions such as walking, movement, and tremor by increasing or substituting the striatal dopamine concentration. These drugs include L-DOPA, dopamine agonists, MAO-B inhibitors, COMT inhibitors, anticholinergics, and amantadine. Although these drugs do not modify the course of the disease or treat nonmotor aspects of PD, they may lead to notable improvements in motor symptoms in the majority of patients, particularly in the early stages of the disease [51]. However, the benefit of the drugs usually diminishes over time and becomes less consistent in controlling symptoms. 

The aforementioned medications may also result in problematic side effects. For example, L-DOPA is delivered to all areas of the brain other than the dopamine-depleted striatum, resulting in side effects such as hallucinations and cognitive impairment in some patients due to the off-target effects of the drug [52]. L-DOPA is often used in combination with a COMT inhibitor that inhibits peripheral levodopa metabolism and thus increases the amount of L-DOPA delivered to the brain [53]. Unfortunately, none of these therapies has been proven to slow the progression of the disease or the emergence of nonmotor- and non–dopamine-associated features. Moreover, significant motor variations and spontaneous movements may develop in patients. For patients exhibiting the second symptom, surgery may be advised. Deep brain stimulation has been well established as a nondestructive treatment commonly used when medical therapy is exhausted for patients with problematic L-DOPA-induced side effects, although this may result in substantial adverse neuropsychiatric effects [54]. Implanted hardware may erode, migrate, or be damaged [55]. Recently, a new method known as magnetic resonance-guided focused ultrasound ablation has been used for the clinical treatment of tremor-predominant PD; it offers a noninvasive and more precise targeting of lesions in thalamic tracts and structures [56].

## 5. Stem Cell-Based Treatments

With better understanding of the etiology and pathogenesis of PD, several important pathways have been revealed as targets for potential treatments. Conventional therapeutic strategies for relieving the symptomatic stages of PD remain, but with new genetic insights it may be possible to use preventive neuroprotective treatments for people at risk of developing PD, thus delaying the onset and progression of the disease. In parallel with efforts to prevent and control symptomatic PD, researchers are also investigating stem cells as replacements for diseased neurons or degenerated tissues [57].

DA cell transplantation is believed to be the most promising cell replacement therapy. Transplanting midbrain DA neurons into DA-depleted striatum can restore DA neurotransmission to replace lost neurons in patients with PD. Some stem cell approaches are being investigated as a potential means of cell replacement through regenerative treatment. Clinical trials revealed that the transplantation of fetal midbrain tissues relieved neurological symptoms and restored motor functions in patients with PD [58,59]. Striatal grafts of midbrain DA neurons can be obtained from various sources, such as fetal tissues, porcine fetal SN neurons, carotid body cells, and immature retinal cells [60]. Although the safety and efficacy of different cell replacement therapies require further investigation in humans, they are speculated to fit within the future scope of PD management.

### 5.1. Fetal Ventral Mesencephalon Tissue

Fetal ventral mesencephalon (VM) consists of distinct neuronal populations including DA neurons of the SN and VT areas, oculomotor neurons, and reticular neurons [61]. In the late 1970s and 1980s, the first open-label clinical trial transplanting fetal VM cells to the brains of PD patients was performed [62,63]. DA neurotransmission recovery initiates at six months, with its progressive restoration suggesting the continuing evolution of the transplanted cells [64]. However, postmortem analysis of patients who had received fetal VM grafts revealed evidence of LB pathology in the transplanted cells in some patients [65,66], leading to the hypothesis that LB pathology may spread from host to graft [67]. The early fetal VM transplantation trials used tissues from surgical terminations of pregnancy, but medical (nonsurgical) terminations are often used in recent clinics [68,69]. 

Extensive testing using fetal VM cells for transplantation in animals has revealed that they can ameliorate the symptoms of PD. Regarding clinical trials, in a European, open-label, assessor-blinded multicenter trial by TRANSEURO, VM tissues were transplanted to 150 patients with predefined desirable characteristics for neural grafting, that is, younger-onset PD without notable levodopa-induced dyskinesia [70]. The patients were clinically observed and regularly assessed with positron emission tomography (PET) and magnetic resonance imaging scans for over four years [71]. The TRANSEURO projects have helped define the preclinical and clinical standards, feasibility, and clinical efficacy of fetal cell therapy, and they have provided protocols for future cell-based therapy using, for example, induced pluripotent stem cell (iPSC)-derived DA neurons [72].

Several technical problems remain before transplantation of fetal VM can be applied in clinical practice. One major limitation is the poor survival of grafted DA neurons and limited dopaminergic reinnervation in the host striatum. Attempts have been made to solve these problems through inclusion of a neurotrophic factor (e.g., brain-derived neurotrophic factor [BDNF]) and cotransplantation with neural/paraneural origins [73]. A second problem is the limited availability of human fetal tissues, and the lack of standardization and the variation in protocols. Transplants of cells other than authentic fetal VM DA neurons have failed to demonstrate key requisite properties, including robust survival, DA release, and clinical benefits [65,74]. Notably and not exclusively for fetal VM transplantation, administration of an immunosuppressant such as cyclosporine is required throughout the transplantation and observational period to prevent allograft-induced immune rejection. Alternative cell therapies using hypoimmunogenic cells such as mesenchymal stem cells (MSCs) have been tested.

### 5.2. MSCs

MSCs have been proven to be beneficial for treating many diseases including PD [75]. As MSCs present low immunogenicity, no teratoma risk, little ethical concern, and a low probability of being tumorigenic after transplantation into the human body [76,77], clinical trials using MSCs as a therapeutic agent are underway (Table 1). 

MSCs can be isolated from various sources, including bone marrow aspirate, adipose tissue, peripheral blood from adult tissues, and Wharton’s jelly of the umbilical cord from neonatal tissues [78,79,80]. Human umbilical cord MSCs are ideal for use in therapeutic application because of their multilineage differentiation capability, autologous transplantation feasibility, easy acquisition, and lack of ethical problems [81]. However, transplanted human umbilical cord MSCs have a low survival rate in the host, and vein-transplanted cells may cause capillary embolization and uncontrollable cell division. Only a small proportion of transplanted human umbilical cord MSCs has been identified in the target tissue [82,83]. Despite this, MSCs from different sources led to improvements in PD based on symptoms in a 6-OHDA mouse model after transplantation. Some improvements seem to depend on MSC-secreted neurotrophic factors (BDNF, glial cell line-derived neurotrophic factor, nerve growth factor) that are able to protect DA neurons from apoptosis and stimulate neurogenesis by releasing mitotic and proangiogenic factors such as fibroblast growth factor 2, endothelial growth factor, and vascular endothelial growth factor [84,85]. 6-OHDA is known to create oxidative stress and trigger neuroinflammation, leading to neuronal damage and dopaminergic denervation [86,87]. The neurotrophic factors secreted by MSCs may stimulate differentiation of the resident stem cells and protect regenerated neurons against stress-induced apoptosis (a neuroprotective effect) [88]. However, the immunomodulatory properties of MSCs also play a role in the inhibition of microglial activation by secreting a number of immunosuppressive cytokines (TGF-β1, PGE2, HGF, IDO, NO, IL-10 and IL-6) or by direct cell–cell contact [89]. 

Under neuronal induction conditions, MSCs can be induced to differentiation toward neuron-like cells and astrocytes. When transplanted into PD animal models, both neuronal-primed MSCs and undifferentiated MSCs revealed a beneficial effect, suggesting that MSCs may not necessarily counteract PD through direct cell replacement. MSCs not only alleviate PD symptoms by their antiapoptotic, anti-inflammatory, and neuroprotective effects in the neurotoxin-induced PD models but may also improve α-syn–induced DA neuron degeneration. In a PD model expressing the A53T α-syn mutant, MSCs stabilized microtubule assembly by inhibiting α-syn–induced tau phosphorylation; therefore, axonal trafficking was increased to facilitate autophagolysosome-dependent clearance of α-syn and prosurvival effects on DA neurons [90].

Although MSCs can be obtained from multiple sources and have fewer ethnical concerns than fetal VM tissues, MSCs are heterogeneous and sometimes difficult to distinguish from fibroblasts [91]. Moreover, MSCs cannot be expanded for many passages. Clinical-grade MSCs usually require MSCs to be transplanted before passage 3; however, the cell number at this passage is usually insufficient for transplantation [92].

### 5.3. Pluripotent Stem Cell-Derived Neuron Progenitor Cells

Pluripotent stem cells (PSCs), such as embryonic stem cells (ESCs) and iPSCs, can be differentiated to diverse cell types including neurons with DA properties. As PSCs can self-renew through unlimited replications, an unlimited number of cells that can be used for neural grafting can be obtained. iPSC-based cell replacement therapy has gained popularity since its discovery by Shinya Yamanaka because iPSCs have a similar differentiation potential to ESCs but fewer ethical concerns [81,93]. 

Human PSCs (hPSCs) are a promising source of cells for regenerative medicine. They are an appealing cell source for replenishing progressively degenerated midbrain DA neurons associated with advanced aging and PD [94]. The directed differentiation of hPSCs into specialized cell types such as spinal motor neurons or DA neurons has been achieved [49]. Protocols for differentiating human ESCs/iPSCs to regionally specified neural subtypes have been well established for both monolayer (2D) and organoid (3D) cultures [95,96]. When hPSCs are cultured under serum-free conditions, they easily differentiate into the neuroectoderm [97]. Dual-SMAD inhibition by Noggin (or LDN193189) and SB431542 in ESCs promotes floor plate (FP) differentiation [98]. WNT activation (achieved by treating the cell with WNT3A or GSK3B inhibitor CHIR99021) in addition to dual-SMAD inhibition further drive midbrain FP toward the ultimate fate of DA neurons by coexpressing FOXA2 and LMX1A. Induction of FOXA2/LMX1A can be further promoted by the activation of SHH signaling. Under a neuronal maturation medium, TH-positive and electrophysiologically active DA neurons were obtained in both 2D and 3D cultures over 80 days of differentiation [94]. 

DA neurons generated using the aforementioned FP-based strategy can be efficiently engrafted in vivo after transplantation and can functionally recover motor deficits in animal models of PD [94]. Behavioral analysis indicated recovery of amphetamine-induced rotation asymmetry [99]. Furthermore, when the DA progenitors were transplanted into the striatum of 6-OHDA PD rats, the animals demonstrated behavioral improvements in tests of forelimb use and akinesia [100,101]. Compared with human fetal DA neurons, hESC-derived midbrain DA neurons revealed comparable effects as assessed by graft survival, functionality, and efficacy in restoration of motor function in PD rats [102].

Long-term engraftment and survival are critical for cell therapy because a single dose of transplantation lowers the risk of neurosurgery. In 6-OHDA-lesioned mice and rats, midbrain DA neurons derived from hESCs were demonstrated to survive longer than six months after transplantation, as evaluated by PET and single photon emission computed tomography [102]. In vivo electrophysiological and neurochemical activities of the graft could be monitored and assessed in real-time owing to the development of optogenetics [103]. To release DA and support functional recovery, precise control over the engrafted cell activity is highly desirable. Tunable rescue of motor function in PD mice was achieved by drug-dependent stimulation or inhibition of grafted human DA neurons [104].

One advantage of using iPSCs for cell replacement therapy is that autologous iPSC-derived cells can be used in the donor’s own body with reduced concerns for immune rejection [105]. The validity of this concept has been shown in nonhuman primates. Transplantation of autologous iPSC-derived DA neurons into MPTP-lesioned cynomolgus monkey brains revealed a marked improvement of motor impairment without immunosuppression [106]. Regarding clinical trials, iPSC-derived DA cells of human origin must be tested on a preclinical nonhuman primate model with longer posttransplantation observations of safety and efficacy. hPSC-derived DA progenitor cells were demonstrated to survive, mature, and function with good outcomes as midbrain DA neurons in an MPTP-induced primate PD model without formation of tumors in the brain for at least two years [107,108]. With these encouraging preclinical results, clinical trials using hESC/iPSC-derived cell products for treating PD have been started in Australia (NCT02452723), China (NCT03119636), and Japan (JMA-IIA00384, UMIN000033564), but have more superior risk management than other clinical trials using MSCs [109].

Other stem cells such as human amniotic epithelial stem cells (hAECs) are also used in clinical trials. These cells can promote neural cell survival and regeneration, repair affected neurons, and synthesize and release neurotrophic factors and neurotransmitters [110,111].

### 5.4. Advantages and Disadvantages of iPSC-Based Therapy

**Figure 2:**
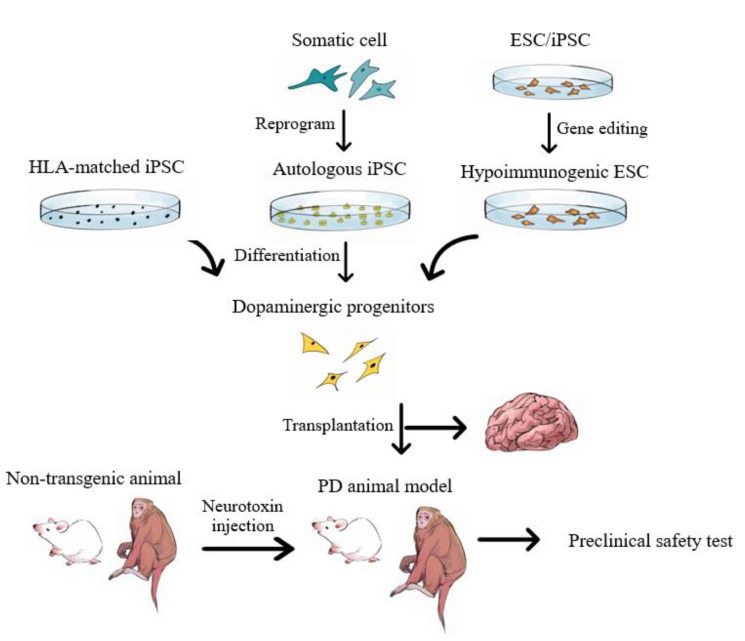
Stem cell therapy for Parkinson disease. Dopaminergic progenitors could be obtained from different sources of pluripotent stem cells, which are either derived from somatic cells by epigenetic reprogramming, or from IVF-derived human embryos. HLA-matched iPSC or gene-edited hyopimmunogenic ESC/iPSC lowers the risk of graft-induced rejection. Preclinical tests in neurotoxin-induced PD models in rodents and nonhuman primates show promising therapeutic effect. IVF: in vitro fertilization; HLA: human leukocyte antigen.

Cell-based therapies including MSCs have been tested preclinically and clinically, and many results have shown them to correct a diseased state by replacing degenerating neurons with normal ones (Figure 2) [87]. The therapeutic benefits, however, may not be sustained for a satisfactory period of time owing to the presence of endogenous pathological proteins that might affect the transplanted functional cells [112]. When α-syn cell–cell transfer occurred, α-syn–containing LBs gradually appeared in grafted neurons [113,114]. 

Both iPSC- and ESC-derived DA neurons can survive after being grafted to animals, growing axons that innervate the brain and support functional recovery from neurotoxin-induced motor deficits. As mentioned in a previous section, generating autologous implants requires potential reprogramming of the patient’s fibroblasts to iPSCs, a prospective advantage of iPSC-derived cells over ESC-derived cells. Using patient iPSC-derived neural grafting products avoids the immunosuppression necessary for the survival of ESC-derived or fetal allografts. 

However, generation of a clinical-grade autologous iPSC line is time consuming and costly, and it may require additional gene editing of the causative genes of patients with inherited familiar PD. To overcome these limitations, generation of hypoimmunogenic and universal iPSC lines from a “healthy” and well-characterized donor may be an option. Schrepfer and colleagues generated iPSC lines with inactivated major histocompatibility complex (MHC) class I and II genes and overexpressed CD47. These modified iPSCs, similar to fetal cells that are immune tolerant to the maternal immune system, are immune privileged and evade immune rejection in MHC-mismatched allogeneic recipients without immunosuppression [115]. A second option in using allogeneic cells is to produce an HLA-matched iPSC line. To match 93% of the UK population, at least 150 selected homozygous HLA-type healthy donors are required to generate a tissue bank for making clinical-grade iPSC lines [116], with matching for 90% and 41% of the Japan and Korean populations, respectively [117,118]. This project would require substantial effort involving a thorough characterization of the numerous PSC lines created. As a proof of principle, CRISPR-mediated knockout of HLA-B in iPSC resulted in reduced immunogenicity [119].

The use of cell therapy products from MHC-matched allogeneic or autologous grafts has recently been challenged by an experiment transplanting autologous and MHC-matched neuronal grafts in a primate model of Huntington disease, but long-term survival of neuronal grafts in lesioned brains remain to be demonstrated [120]. Therefore, we still need to be cautious when moving human PSC-derived products from bench to clinics, and other safety issues such as the potential tumorigenicity of iPSC lines, the heterogeneity and purity of cell products, genetic variations, and aberrant epigenetic disease-causing memory in the grafts remain to be resolved.

## 6. Future Prospects

Current treatments for PD focus on symptomatic management through restoration of DA activity but do not address progressive neurodegeneration. A clear medical need for disease-modifying therapies exists. Stem cell research may lead to the development of radical new therapies that have been proposed as new approaches for several neurodegenerative diseases currently lacking effective treatments. Remarkable progress has been made in the development of approaches for generating the types of human-derived neurons and glial cells needed for cell replacement therapy based on pathology for the respective diseases. The mechanisms of stem cells and their progeny underlying behavioral recovery in animal models are better understood than they were a few years ago. Aside from cell replacement, stromal cells such as MSCs are known to lead to improvements that could also be of clinical value through immunomodulation, trophic actions, neuroprotection, and stimulation of angiogenesis. These advancements in stem cell research may lead to a large number of scientifically justified clinical trials in neurodegenerative diseases within the next couple of years.

Strategies for stem cell-based therapy and cell replacement require the implantation of autologous or allogeneic grafts into the diseased brain. Despite improvements in tissue isolation, cell preparation, reprogramming, and differentiation optimizations, the risks of cell and environmental contamination remain. Therefore, if we were able to reprogram the abundant nonneuronal cells in the brain such as astrocytes and glial cells and convert them into functional neurons, this would solve the above problems associated with cell transplantation. Mouse brain astrocytes can be converted to such ‘induced dopamine-releasing’ neurons that can partially modify motor defects [121]. The concept of in vivo reprogramming has been proposed and recently demonstrated by Xiang-Dong Fu’s laboratory team: their team demonstrated that DA neurons could be induced by RNA-binding protein PTB (polypyrimidine tract-binding protein 1) depletion in midbrain astrocytes, therefore potently restoring striatal DA neurons and promoting reconstitution of the nigrostriatal circuit. Correction of motor impairment phenotypes was observed in a chemically induced PD mouse model [122,123]. One advantage of this approach is its simplicity, because transient suppression of PTB by antisense oligonucleotides is sufficient to reverse the PD phenotype. In the future, we foresee the development of new drugs that may specifically target PTB in astrocytes as well as combined treatments using different stem cells and stem cell-derived products. In vivo gene editing may add another avenue for treating familiar PD by correcting characterized recessive mutations.

## 7. Conclusions

Preclinical studies have demonstrated the vast potential of cell replacement therapy for treating neurodegenerative diseases such as PD. Protocols for the derivation of DA precursors are well established and characterized, giving rise to a large number of clinically relevant cells. This review has focused on the advantages and potentials of stem cell therapy. However, many questions and obstacles exist for stem cell therapy. Nowadays, the primary challenges limiting the clinical uses of stem cells involve ethical questions, tumorigenesis, immune response, and, to some degree, toxicity. Considerable ethical issues exist around using hESC, and it produces intense immune responses. Compared with ESCs, iPSCs have fewer ethical issues and reduced immune rejection, but because of their powerful pluripotency, the risk of tumor development may be greater than with other stem cells. Furthermore, iPSCs derived from patients with PD may carry pathological gene mutations that affect the prognosis for cell replacement therapy. To obtain a higher rate of survival and integration, the details of canonical grafting procedures must be stimulated with more studies. In short, stem cell therapy is currently at an initial stage, and great efforts are required to move it forward. For nonmotor improvements, we require new experimental systems for developing symptomatic therapies.

In summary, although we are not yet examining a disease-modifying treatment, stem cell transplantation has the potential to be at the forefront of such PD treatments in the future. The transplantation process and the procedures required for its optimization are still not fully understood, and further research is required to achieve treatment for PD.

## Figures and Tables

**Table 1:** Clinical trials using stem cells as treatments for PD.

Target	Action	Agent (Country)	Trial Phase	Clinical Trial Identifier	Year for Starting	Status
Human neural stem cells	Human fetal neural stem cell injection delivered nasally	Second Affiliated Hospital of Soochow University (China)	Phase 2/3	NCT03128450	2017	Unknown
	Human parthenogenetic neural stem cells injected intracerebrally to the striatum and substantia nigra	Cyto Therapeutics Pty Limited (Australia)	Phase 1	NCT02452723	2015	Active, not recruiting
	Differentiated neurons derived from adult central nervous system progenitor cells transplanted in patients. Stereotactic delivery of cell suspension into basal ganglia structures	NeuroGeneration	Phase 1/2	NCT03309514	2017	Not yet recruiting
Mesenchymal stem cells	Umbilical cord-derived MSC from mesoderm possesses strong proliferation ability and multiple differentiation potentials, delivered by intravenous infusion to patients with PD	Guangzhou General Hospital of Guangzhou Military Command (China)	Phase 1	NCT03550183	2018	Enrolling by invitation
	Allogenic umbilical cord-derived stem cells injected intravenously to enrolled patients with PD	University of Jordan (Jordan)	Phase 1/2	NCT03684122	2018	Recruiting
	Phase IIa double-blind randomized placebo-controlled trial	The University of Texas Health Science Center (USA)	Phase 2	NCT04506073	2020	Not yet recruiting
Human stem cells (OK99)	Implantation of Celavie human stem cells (OK99) to address the underlying pathology of the disease by replacing damaged/destroyed cells of the brain and stimulating the patient’s brain to repair itself	Celavie Bioscences, LLC (Mexico)	Phase 1	NCT02780895	2016	Unknown
Human amniotic epithelial stem Cells (hAESCs)	Stereotactic transplantation of hAESCs into the lateral ventricle	Shanghai East Hospital (China)	Early Phase 1	NCT04414813	2020	Not yet recruiting
Human embryonic stem cells	Transplantation of human embryonic stem cell-derived neural precursor cells into the striatum.	Chinese Academy of Sciences (China)	Phase 1/2	NCT03119636	2017	Recruiting
Induced pluripotent stem cells	Develop human-induced pluripotent stem cells from cell cultures taken from skin biopsies or patients’ hair	Hadassah Medical Organization (Israel)	Not Applicable	NCT00874783	2009	Recruiting
	Total dose of iPSC-derived neural stem cells administered on day 0	Allife Medical Science and Technology Co. Ltd. (China)	Early Phase 1	NCT03815071	2019	Not yet recruiting
Bone marrow–derived stem cells	Isolation of autologous bone marrow-derived stem cells and transfer to the vascular system and interior third of the nasal passages	MD Stem Cells (USA)	Not Applicable	NCT02795052	2016	Terminated
	Autologous bone marrow-derived stem cells stereotactically transplanted into the patient’s striatum	Jaslok Hospital and Research Centre (India)	Not Applicable	NCT00976430	2009	Recruiting
	Allogeneic bone marrow-derived MSCs delivered intravenously	The University of Texas Health Science Center (USA)	Phase 1/2	NCT02611167	2015	Completed
